# Two novel species of *Neoaquastroma* (Parabambusicolaceae, Pleosporales) with their phoma-like asexual morphs

**DOI:** 10.3897/mycokeys.34.25124

**Published:** 2018-05-23

**Authors:** Chayanard Phukhamsakda, Darbhe J. Bhat, Sinang Hongsanan, Jian-Chu Xu, Marc Stadler, Kevin D. Hyde

**Affiliations:** 1 Center of Excellence in Fungal Research, Mae Fah Luang University, Chiang Rai 57100, Thailand; 2 Key Laboratory for Plant Diversity and Biogeography of East Asia, Kunming Institute of Botany, Chinese Academy of Science, Kunming 650201, Yunnan, China; 3 Formerly, Department of Botany, Goa University, Goa, India; No. 128/1-J, Azad Housing Society, Curca, Goa Velha, India; 4 Germplasm Bank of Wild Species, Kunming Institute of Botany, Chinese Academy of Science, Kunming 650201, Yunnan, China; 5 Centre of Mountain Ecosystem Studies, Kunming Institute of Botany, Chinese Academy of Sciences, Kunming 650201, Yunnan, China; 6 Department of Microbial Drugs, Helmholtz Centre for Infection Research, Braunschweig, Germany

**Keywords:** Dothideomycetes, holomorph, Massarineae, saprotrophs, Southeast Asia

## Abstract

The monotypic genus *Neoaquastroma* (Parabambusicolaceae, Pleosporales) was introduced for a microfungus isolated from a collection of dried stems of a dicotyledonous plant in Thailand. In this paper, we introduce two novel species, *N. bauhiniae* and *N. krabiense*, in this genus. Their asexual morphs comprise conidiomata with aseptate and hyaline conidia. *Neoaquastroma bauhiniae* has ascomata, asci and ascospores that are smaller than those of *N. krabiense*. Descriptions and illustrations of *N. bauhiniae* and *N. krabiense* are provided and the two species compared with the type species of the genus, *N. guttulatum*. Evidence for the introduction of the new taxa is also provided from phylogenetic analysis of a combined dataset of partial LSU, SSU, ITS and *tef1* sequence data. The phylogenetic analysis revealed a distinct lineage for *N. bauhiniae* and *N. krabiense* within the family Parabambusicolaceae.

## Introduction

Thailand is a highly biodiverse country in the tropics with hot and humid climate (MacKinnon et al. 1986, Marod and Kutintara 2012). Although the fungal diversity in Thailand has been relatively well-studied (Rostrup 1902, Schumacher 1982, Hyde 1989, Jones 2000, Jones et al. 2006, Suetrong et al. 2009), the number of species being discovered is steadily growing due to increasing activities in studying microfungi in a large variety of terrestrial and aquatic ecosystems (Mapook et al. 2016, Phukhamsakda et al. 2016, Dai et al. 2017, Doilom et al. 2017, Phukhamsakda et al. 2017).

The family Parabambusicolaceae was introduced for a distinct phylogenetic lineage in the suborder Massarineae (Pleosporales) (Tanaka et al. 2015). Species of Parabambusicolaceae are characterised by pseudothecioid ascomata with or without stromatic tissues, papillate to apapillate ostioles, clavate to fusiform asci and hyaline or brown phragmospores (Liu et al. 2015, Tanaka et al. 2015, Li et al. 2016, Wanasinghe et al. 2017). The asexual morphs are sporodochial or *Monodictys*-like (Tanaka et al. 2015, Ariyawansa et al. 2015). Currently, there are seven known genera in this family; *Aquastroma*, *Monodictys*-like spp., *Multilocularia*, *Multiseptospora*, *Neoaquastroma*, *Parabambusicola* (with *P. bambusina* as generic type) and *Pseudomonodictys* (Tanaka and Harada 2003, Wijayawardene et al. 2017, 2018). The genus *Neoaquastroma* Wanas., E.B.G. Jones & K.D. Hyde, has been introduced from a dead twig of a herbaceous plant collected in Northern Thailand and been typifed with *N. guttulatum* Wanas., E.B.G. Jones & K.D. Hyde (Wanasinghe et al. 2017). The genus is characterised by immersed, glabrous pseudothecia, short, papillate, fissitunicate, clavate asci and ellipsoidal to subfusiform, multi-septate hyaline phragmospores, surrounded by a mucilaginous sheath (Wanasinghe et al. 2017). Molecular phylogenetic analysis using ribosomal DNA (LSU, SSU and ITS) and translation elongation factor 1-alpha (*tef1*) sequence data support it as a distinct genus in Parabambusicolaceae.

The purpose of this study is to describe two new species of *Neoaquastroma* from collections of dicotyledonous plants in Thailand. Phylogenetic analysis of combined of LSU, SSU, ITS and *tef1* sequence data are provided.

## Materials and methods

### Sample collection, morphological study and isolation

Fresh specimens were collected from northern and southern part of Thailand during 2015–2017. The specimens were packed into brown paper bags for transport to the laboratory. Pure cultures were obtained from single ascospores on malt extract agar (MEA; 62 g/l) in distilled water following the method of Chomnunti et al. (2014). Cultures were incubated at 25 °C for up to 8 weeks. Induction of asexual reproduction has been adapted from Tanaka and Harada (2003) by placing agar squares with mycelia on water agar placed with sterile rice straw pieces. The plates were incubated at room temperature (25 °C) with the standard light cycles, 12 hrs in the light followed by 12 hrs in the dark for about eight weeks until the fructifications were produced. Type specimens are deposited in Mae Fah Luang University (MFLU) herbarium and isotypes are deposited at the Kunming Institute of Botany, Academia Sinica Herbarium (HKAS), China. Ex-type living cultures are deposited at the Mae Fah Luang Culture Collection (MFLUCC) and duplicates at the International Collection of Microorganisms and Plants (ICMP), New Zealand. Faces of fungi numbers (Jayasiri et al. 2015) and MycoBank number (http://www.MycoBank.org) are provided. Samples were examined under a Nikon ECLIPSE 80i compound microscope and photographed with a Canon 600D digital camera fitted to the microscope. Measurements were made using Tarosoft (R) Image Frame Work programme and photo-plates were made by using Adobe Photoshop CS6 Extended version 10.0 software (Adobe Systems, United States).

### DNA extraction, amplification and sequencing

DNA was extracted from mycelium by using Biospin Fungus Genomic DNA Extraction Kit (BioFlux) (Hangzhou, P. R. China) and gene extraction kit (Bio Basic Inc., Canada). PCR amplification was carried out using primers LROR/LR5 for the nuclear ribosomal large subunit 28S rDNA gene (LSU), NS1/NS4 for the nuclear ribosomal small subunit 18S rDNA gene (SSU) and ITS5/ITS4 for internal transcribed spacer rDNA region (ITS1, 5.8S rDNA and ITS2); partial fragments of the translation elongation factor 1-alpha (*tef1*) gene region was amplified using primers EF1-983F and EF1-2218R (Vilgalys and Hester 1990, White et al. 1990, Carbone and Kohn 1999). Primer sequences are available at the WASABI database at the AFTOL website (aftol.org). Amplification reactions for LSU, SSU and ITS followed Phukhamsakda et al. (2016). The PCR thermal cycle programme for EF1-983F and EF1-2218R (Carbone and Kohn 1999) for translation elongation factor 1-alpha (*tef1*) was set for denaturation at 96 °C for 2 min, followed by 40 cycles of denaturation at 96 °C for 45 sec, annealing at 54 °C for 30 sec and extension at 72 °C for 1.30 min, with a final extension step at 72 °C for 5 min. Genomic DNA and PCR amplification products were checked on 1% agarose gel. PCR products were purified as described in Wendt et al. (2017), sequences were generated by Shanghai Sangon Biological Engineering Technology & Services Co. (Shanghai, P.R. China) and sequencing services at Helmholtz Centre For Infection Research (HZI, Braunschweig, Germany).

### Sequence alignment and phylogenetic analysis

SeqMan v. 7.0.0 (DNASTAR, Madison, WI) was used to assemble consensus sequences. Sequences of closely related strains were retrieved using BLAST searches against GenBank (http://www.ncbi.nlm.nih.gov). We also included the strains from Wanasinghe et al. (2017) and these are listed in Table 1. Sequences were aligned with MAFTT version 7.220 (Katoh et al. 2013) online sequence alignment tools (mafft.cbrc.jp/alignment/server), with minimal adjustment of the ambiguous nucleotides by visual examination and manually corrected in AliView programme (Larsson 2014). Leading or trailing gaps exceeding from primer binding site were trimmed from the alignments prior to tree building and alignment gaps were treated as missing data. The concatenation of the multigene alignment was created in MEGA 6 (Tamura et al. 2013).

Maximum likelihood analyses (ML), including 1,000 bootstrap replicates, was performed using RAxML (Stamatakis 2014) as implemented in raxmlGUI version v.1.3.1 (Silvestro and Michalak 2011). The search strategy was set to rapid bootstrapping. The analysis was carried out with the general time reversible (GTR) model for nucleotide substitution and a discrete gamma-distributed with four rate categories (O’Meara et al. 2006, Stamatakis et al. 2008). The bootstrap replicates were summarised on to the best scoring tree.

The best fitting substitution model for each single gene partition and the concatenated data set was determined in MrModeltest 2.3 (Nylander 2004) for Bayesian inference posterior probabilities (PP). In our analysis, GTR+I+G model was used for each partition. The Bayesian inference posterior probabilities (PP) distribution (Zhaxybayeva and Gogarten 2002) was estimated by Markov Chain Monte Carlo sampling (MCMC) in MrBayes v. 3.2.2 (Huelsenbeck and Ronquist 2001). Six simultaneous Markov chains were run for 1,000,000 generations and trees were sampled every 100^th^ generation, thus 10,000 trees were obtained. The suitable burn-in phases were determined by traces inspected in Tracer version 1.6 (Rambaut et al. 2014). Based on the tracer analysis, the first 1,000 trees representing 10% of burn-in phase of the analyses were discarded. While the remaining trees were used for calculating posterior probabilities in the majority rule consensus tree (critical value for the topological convergence diagnostic set to 0.01).

**Figure 1.**
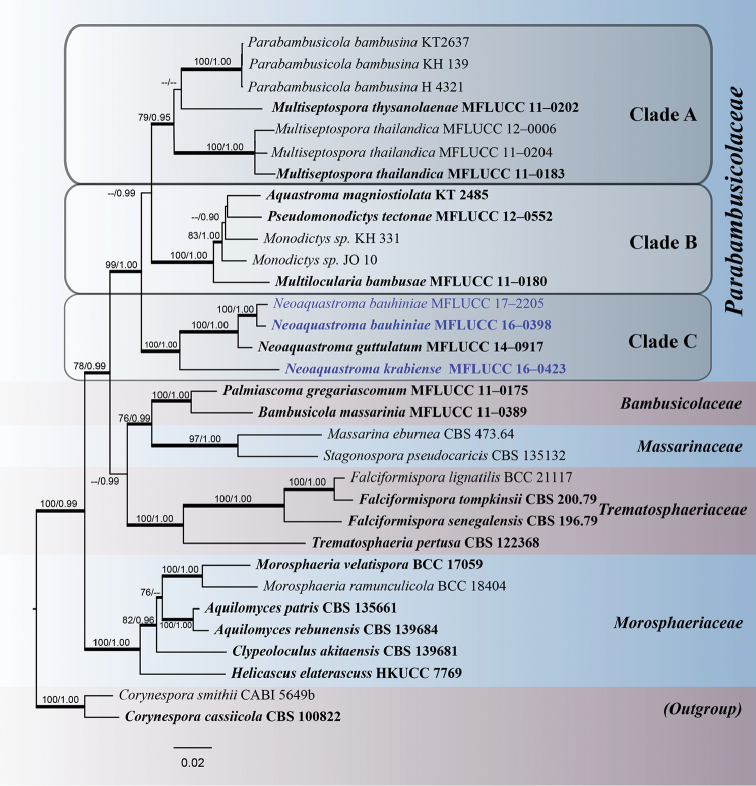
The best scoring RAxML tree based on a combined partial LSU, SSU, ITS and *tef1* gene datasets. Bootstrap values (BS) from maximum likelihood (ML, left) of more than 70% BS and Bayesian posterior probabilities (PP, right) greater than 0.90 are given above or below the nodes. The tree is rooted with *Corynespora smithii* (CABI 5649b) and *C. cassiicola* (CBS 100822) in Corynesporaceae. The species, determined in this study, are indicated in blue. The ex-type and references strains are indicated in bold. Hyphens (-) represent support values less than 70% BS/0.90 PP. Thick branches represent significant support values from all analyses (BS ≥ 70%/PP ≥ 0.95).

Phylogenetic trees and data files were visualised in FigTree v. 1.4 (Rambaut and Drummond 2008). The phylogram with bootstrap values and/or posterior probabilities on the branches are presented in Fig. 1 by using graphical options available in Adobe Illustrator CS v. 6. All sequences generated in this study were submitted to GenBank. The finalised alignment and tree were deposited in TreeBASE, submission ID: 22419 (http://www.treebase.org/). Maximum likelihood bootstrap values equal to or greater than 70% with Bayesian Posterior Probabilities (PP) equal or greater than 0.90 are presented below or above each node (Fig. 1).

## Results

### Phylogenetic analyses

The phylogenetic tree included 32 taxa representing six families from the suborder Massarineae. The phylogenetic trees from each individual data sets were initially generated, these were not significantly different (data not shown) and therefore combined data sets were performed. The combined dataset consisting 3,554 nucleotide characters, of which 1,001 characters corresponded to LSU, 1,038 characters to SSU, 508 characters to ITS and 929 characters to *tef1*. *Corynespora smithii* (CABI 5649b) and *C. cassiicola* (CBS 100822) are used as outgroup taxa. An insertion in the SSU rDNA region of isolates *Aquilomyces rebunensis* Tanaka & K. Hiray. (CBS 139684), *Clypeoloculus akitaensis* Tanaka & K. Hiray. (CBS 139681) and *Trematosphaeria pertusa* Fuckel (CBS 122368) were excluded from the analysis prior to tree building. The best scoring tree from maximum likelihood analysis was selected with a final likelihood value of – In: 23014.934293 and the result is presented in Fig. 1. Phylogenetic trees obtained from maximum likelihood and Bayesian analyses yielded trees with similar overall topology as that of previous work (Tanaka et al. 2015, Liu et al. 2015, Wanasinghe et al. 2017).

In this study, the family Parabambusicolaceae received high support in the phylogenetic analysis. While within the family, the taxa are separated into three subclades (Fig. 1). *Parabambusicola bambusina*, the generic type, clustered with *Multiseptospora* with high support. However, *M. thysanolaenae* (MFLUCC 11-0202) formed a sister taxon with *Parabambusicola bambusina* (Clade A). *Aquastroma magniostiolata* (CBS 139680) and *Multilocularia bambusae* (MFLUCC 11-0180) formed a clade with the hyphomycetes strains of *Monodictys* spp. and *Pseudomonodictys tectonae* (MFLUCC 12-0552) with high support in all computational methods (Clade B). *Neoaquastroma* formed a basal clade (Clade C), with *N. bauhiniae* (MFLUCC 16-0398, 17-2205) and *N. krabiense* (MFLUCC 16-0423) clustered with the type species *N. guttulatum*, with strong support (100% ML /1.00 PP). We describe the new taxa based on agreement in support for all computational methods (Jeewon and Hyde 2016). The new sequence data is deposited in GenBank (Table 1).

**Table 1.** Taxa used in the phylogenetic analysis and their corresponding culture collections, and accession numbers used in this study.

Taxon	Culture accession number(s)^1,2^	GenBank accession numbers				References
		LSU	SSU	ITS	*tef1*	
*Aquastroma magniostiolata*	**CBS 139680^T^ = MAFF 243824**	AB807510	AB797220	LC014540	AB808486	Tanaka et al. 2015
*Aquilomyces patris*	**CBS 135661^T^**	KP184041	KP184077	KP184002	–	Knapp et al. 2015
*Aquilomyces rebunensis*	**CBS 139684^T^**	AB807542	AB797252	AB809630	AB808518	Tanaka et al. 2015
*Bambusicola massarinia*	**MFLUCC 11-0389^T^**	JX442037	JX442041	NR_121548	–	Dai et al. 2012
*Clypeoloculus akitaensis*	**CBS 139681^T^**	AB807543	AB797253	AB809631	AB808519	Tanaka et al. 2015
*Corynespora cassiicola*	**CBS 100822^T^**	GU301808	GU296144	–	GU349052	Schoch et al. 2009
*Corynespora smithii*	CABI 5649b	GU323201	–	FJ852597	GU349018	Schoch et al. 2009
*Falciformispora lignatilis*	BCC 21117	GU371826	GU371834	KF432942	GU371819	Schoch et al. 2009
*Falciformispora senegalensis*	**CBS 196.79^T^**	KF015631	KF015636	KF015673	KF015687	Ahmed et al. 2014
*Falciformispora tompkinsii*	**CBS 200.79^T^**	KF015625	KF015639	NR_132041	KF015685	Ahmed et al. 2014
*Helicascus elaterascus*	A22-5A = HKUCC 7769	AY787934	AF053727	–	–	Tanaka et al. 2015
*Massarina eburnea*	CBS 473.64	GU301840	GU296170	–	GU349040	Zhang et al. 2009
*Monodictys* sp.	KH 331 = MAFF 243826	AB807553	AB797263	–	AB808529	Tanaka et al. 2015
*Monodictys* sp.	JO 10 = MAFF 243825	AB807552	AB797262	–	AB808528	Tanaka et al. 2015
*Morosphaeria ramunculicola*	BCC 18404	GQ925853	GQ925838	–	–	Suetrong et al. 2009
*Morosphaeria velatispora*	**BCC 17059^T^**	GQ925852	GQ925841	–	–	Suetrong et al. 2010
*Multilocularia bambusae*	**MFLUCC 11-0180^T^**	KU693438	KU693442	KU693446	–	Li et al. 2016
*Multiseptospora thailandica*	**MFLUCC 11-0183^T^**	KP744490	KP753955	KP744447	–	Liu et al. 2015
*Multiseptospora thailandica*	MFLUCC 11-0204	KU693440	KU693444	KU693447	KU705659	Liu et al. 2015
*Multiseptospora thailandica*	MFLUCC 12-0006	KU693441	KU693445	KU693448	KU705660	Liu et al. 2015
*Multiseptospora thysanolaenae*	**MFLUCC 11-0238^T^**	KU693439	KU693443	–	KU705658	Li et al. 2016
***Neoaquastroma bauhiniae***	**MFLUCC 16-0398^T^**	**MH023319**	**MH023315**	**MH025952**	**MH028247**	**This study**
***Neoaquastroma bauhiniae***	**MFLUCC 17-2205**	**MH023320**	**MH023316**	**MH025953**	**MH028248**	**This study**
***Neoaquastroma krabiense***	**MFLUCC 16-0419^T^**	**MH023321**	**MH023317**	**MH025954**	**MH028249**	**This study**
*Neoaquastroma guttulatum*	**MFLUCC 14-0917^T^**	KX949740	KX949741	KX949739	KX949742	Wanasinghe et al. 2017
*Palmiascoma gregariascomum*	**MFLUCC 11-0175^T^**	KP744495	KP753958	KP744452	–	Liu et al. 2015
*Parabambusicola bambusina*	KH 139 = MAFF 243823	AB807537	AB797247	LC014579	AB808512	Tanaka et al. 2015
*Parabambusicola bambusina*	H 4321 = MAFF 239462	AB807536	AB797246	LC014578	AB808511	Tanaka et al. 2015
*Parabambusicola bambusina*	KT 2637 = MAFF 243822	AB807538	AB797248	LC014580	AB808513	Tanaka et al. 2015
*Pseudomonodictys tectonae*	MFLUCC 12-0552	KT285573	KT285574	–	KT285571	Ariyawansa et al. 2015
*Stagonospora pseudocaricis*	CBS 135132	KF251762	–	KF251259	–	Quaedvlieg et al. 2013
*Trematosphaeria pertusa*	**CBS 122368^ET^**	FJ201990	FJ201991	NR_132040	KF015701	Zhang et al. 2008

^1^ Abbreviations: **BCC**: BIOTEC Culture Collection, Bangkok, Thailand; **CABI**: Centre for Agriculture and Biosciences International, Egham, UK; **CBS**: CBS-KNAW Collections, Westerdijk Fungal Biodiversity Institute, Utrecht, The Netherlands; **CPC**: Culture collection of Pedro Crous, housed at CBS; **HKUCC**: The University of Hong Kong Culture Collection; **HHUF**: Herbarium of Hirosaki University, Fungi; **JCM**: The Japan Collection of Microorganisms, Japan; **JK**: J. Kohlmeyer; **JO**: J. Onodera; **KH**: K. Hirayama; **KT**: K. Tanaka; **MAFF**: Ministry of Agriculture, Forestry and Fisheries, Japan; **MFLU**: Mae Fah Luang University herbarium, **MFLUCC**: Mae Fah Luang University Culture Collection, Chiang Rai, Thailand.

^2^ Status of the strains: (T) ex-type, (ET) ex-epitype. The strains generated in this study are given in bold.

### Taxonomy

#### Neoaquastroma bauhiniae

Taxon classificationFungiPleosporalesParabambusicolaceae

C. Phukhams. & K.D. Hyde sp. nov.

824673

Figure 2

##### Etymology.

Name refers the host from which this fungus was isolated.

##### Type material.

THAILAND. Phrae Province: Song District, on dead twigs of *Bauhinia variegata* L. (Fabaceae), 25 July 2015, C. Phukhamsakda, S1-11, MFLU 17-0002 (**holotype**), MFLUCC 16-0398 = ICMP 21572 (**ex-type living culture**).

##### Description.

*Saprobic* on dead twigs of *Bauhinia variegata* L. *Sexual morph*. *Ascomata* 113–190 μm high × 170–307 μm diam. (x̄ = 160 × 260 μm, n = 10), semi-immersed to immersed, solitary, scattered, subglobose to compressed, coriaceous, brown to dark brown, rough-walled, with short hyphae projecting from peridium, ostiolate. *Ostiole* 33 × 85 μm diam., centrally located, papillate, periphysoid. *Peridium* 8–25 μm wide (x̄ = 17, n = 30), with cells 3–8 μm wide, composed of 3 layers of reddish-brown to dark brown, cells of *textura angularis*, inner layer composed of hyaline gelatinous cells. *Hamathecium* composed of numerous, dense, long, 1–2.4 μm (x̄ = 1.7 μm, n = 50), narrow, filiform, transversely septate, branched, anastomosing, cellular psedoparaphyses. *Asci* 53–116 × 26–43 μm (x̄ = 98 × 37 μm, n = 30), 8-spored, bitunicate, fissitunicate, oboviod to oblong, with furcate pedicel, with ocular chamber visible when immature. *Ascospores* 37–46 × 9–16 μm (x̄ = 43 × 13 μm, n = 50), bi-seriate or overlapping, broad fusiform, narrow towards the apex, initially hyaline, becoming brown to dark brown at maturity, 4–7-transversely euseptate, constricted at the septa, with cell above central septum wider, rough-walled, indentations present, surrounded by 7–12 μm wide, mucilaginous sheath. *Asexual morph* coelomycetous. Pycnidia produced on mycelium in water agar. *Conidiomata* 33−49 μm high × 92–108 μm wide diam., pycnidial, dark brown to black, covered by dense vegetative hyphae, globose, in agar immersed to superficial, uniloculate, solitary to scattered, ostiolate. *Conidiomatal wall* thin, brown to black-walled with cells of *textura angularis*. *Conidiophores* reduced to conidiogenous cells. *Conidiogenous cells* 3−4 × 2−3.5 μm, enteroblastic, phialidic, integrated, oblong, hyaline, formed from the inner layer of pycnidium wall. *Conidia* 2–4 × 1.5–2 μm (x̄ = 3 × 1.7 μm, n = 100), broad-oblong to oval, hyaline, aseptate, smooth-walled.

##### Culture characteristics.

Colonies on MEA, reaching 50 mm diam. after 4 weeks at 25 °C. Culture dark olive-green with black centre, with dense mycelia, circular, flat, umbonate, rough surface, dull, fimbriate, radially furrowed, covered with white aerial mycelium; mycelium strongly radiating into agar, yellow pigment diffusing in the agar; reverse black with radiating brown mycelium. Sexual and asexual morphs formed in culture. Morphology of sexual phase similar to those on substrate.

##### Additional material examined.

THAILAND, Phrae Province, Song District, on dead twigs of *Bauhinia variegata* L. (Fabaceae), 25 July 2015, C Phukhamsakda, S1-11 (**isotype in HKAS**, under the code of HKAS 99513); *ibid*., on dead twigs of *Bauhinia purpurea* L. (Fabaceae), 5 May 2016, C Phukhamsakda, S1_03_16, ex-paratype living culture, MFLUCC 17-2205.

##### Distribution.

Phrae Province, Thailand.

##### Notes.

**Figure 2.**
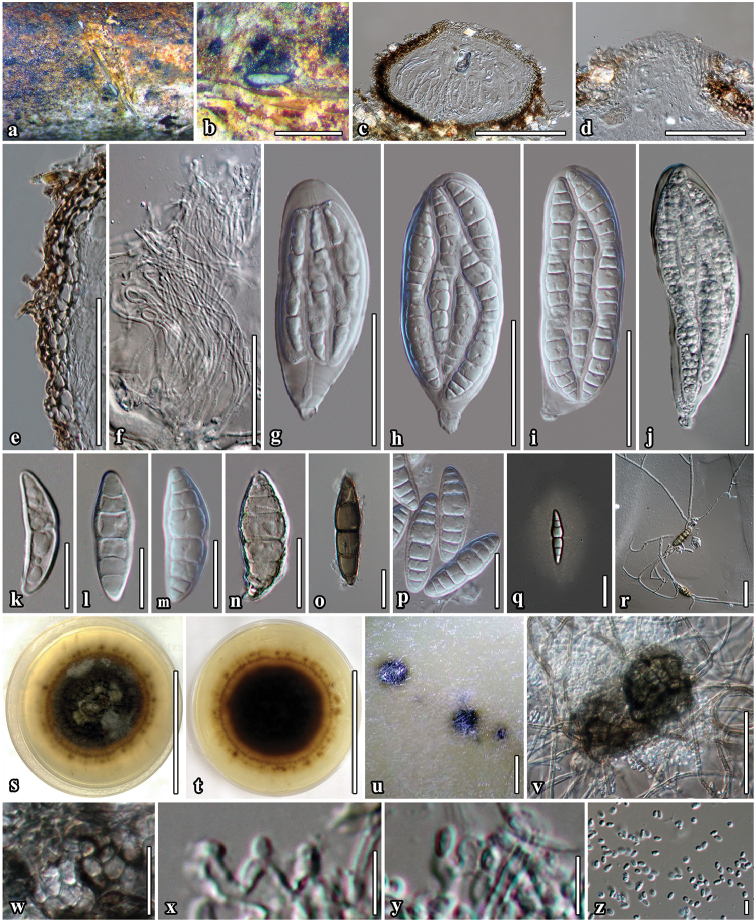
***Neoaquastroma bauhiniae*** (MFLU 17-0002, holotype) **a** Appearance of ascomata on host surface **b** Close up of ascoma **c** Section of ascoma **d** Ostiolar canal **e** Section of partial peridium layer **f** Pseudoparaphyses **g–j** Development state of asci **j** Asci produced in culture **k–p** Development state of ascospores; (**n, o** Senescent spores **m, p** ascospores in 5% of KOH reagent); **q** Ascospores stained with India ink, sheath surrounding the entire ascospore **r** Germinated ascospore **s, t** Culture character on MEA **u** Conidiomata forming on agar on rice straw media after 8 weeks **v** Immature conidiomata **w** Conidiomatal wall **x, y** Conidiogenous cells and developing conidia **z** Conidia **j, m** Asci and ascospore in culture (on rice straw). Scale bars: 500 µm (**b**); 100 µm (**c, v**); 50 µm (**d–j**); 20 µm (**k–r, w**); 5 µm (**x–z**).

*Neoaquastroma bauhiniae* is similar to *N. krabiense*, but the ascomata, asci and ascospores are smaller and the species also has a thinner peridium with 4−7 septate hyaline ascospores. Thus, *Neoaquastroma bauhiniae* is introduced as a second species in *Neoaquastroma* based on its unique morphology coupled with high support values from the phylogenetic analysis (100% ML/1.00 PP, Fig. 1). Tanaka et al. (2015) only described the asexual morph in *Parabambusicola* to produce spermatia. We now obtained a single spore isolate which produces both sexual and asexual morphs in culture. The asexual morph of *Neoaquastroma bauhiniae* produced pycnidial conidiomata with hyaline conidia (Fig. 2, u–z).

#### Neoaquastroma krabiense

Taxon classificationFungiPleosporalesParabambusicolaceae

C. Phukhams. & K.D. Hyde sp. nov.

824674

Figure 3

##### Etymology.

Name refers the location where this fungus was collected.

##### Type material.

THAILAND, Krabi Province: Meuang district, on dead twigs of *Barringtonia acutangula* (Lecythidaceae), 16 December 2015, C. Phukhamsakda, Kr015, MFLU 17-0003 (**holotype**), MFLUCC 16-0419 = ICMP 21572 (**ex-type living culture**).

##### Description.

*Saprobic* on dead twigs of *Barringtonia acutangula* (L.) Gaertn. *Sexual morph*. *Ascomata* 404–498 μm high × 290–319 μm diam. (x̄ = 426 × 300 μm, n = 10), immersed in bark, solitary, scattered or sometimes gregarious, compressed globose, with a flattened base, coriaceous, black to dark brown, smooth, papillate, ostiolate. *Ostiole* 137–146 μm high × 117–154 μm diam. (x̄ = 143 × 137 μm, n = 10), centrally located, oblong, filled with hyaline periphysoid. *Peridium* 45–73 μm wide (x̄ = 56, n = 30), cell width 3–12 (x̄ = 8 μm, n = 40) composed of 6–10(–13 at base) layers of blackish-brown to dark brown, with cells of *textura angularis*, outer layer heavily pigmented, inner layer composed of hyaline gelatinous cells. *Hamathecium* composed of numerous, dense, long, 1.6–2.4 μm (x̄ = 2 μm, n = 50), broad, filiform, transversely septate, branched, anastomosing, cellular pseudoparaphyses. *Asci* 95–169 × 29–45 μm (x̄ = 135 × 35 μm, n = 25), 8-spored, bitunicate, fisitunicate, oboviod to clavate, with furcate pedicel, ocular chamber clearly visible when immature. *Ascospores* 50–64 × 9–18 μm (x̄ = 57 × 13 μm, n = 50), bi-seriate or overlapping, fusiform, narrow towards the apex, hyaline, 5–8-transversely septate, constricted at the septa, cell above central septum slightly wider, rough-walled, indentations present when mature, granulate when stained with India ink, surrounded by 3–9 μm wide, mucilaginous sheath. *Asexual morph* coelomycetous, formed on rice straw agar. *Conidiomata* 84−90 μm high × 73–89 μm wide., pycnidial, uniloculate, confluent or scattered, superficial, covered with dense vegetative hyphae, globose, dark brown to black. *Conidiomatal wall* thin, brown to black-walled cells of *textura angularis*. *Conidiophores* reduced to conidiogenous cells. *Conidiogenous cells* 3−5 × 1.5−4 μm, enteroblastic, phialidic, integrated, broad-cylindrical to oblong, hyaline, formed from the inner layer of pycnidium wall. *Conidia* 2–4 × 1.5–2.5 μm (x̄ = 3 × 2 μm, n = 60), ellipsoidal to oblong, hyaline, aseptate, smooth-walled.

##### Culture characteristics.

Colonies on MEA, reaching 50 mm diam. after four weeks at 25 °C. Culture grey, becoming dark-olive brown after four weeks, of dense mycelia, colonies circular, flat, umbonate, raised from the agar in the centre, surface rough, dull, covered with aerial mycelium, white mycelium radiating into the agar, pale orange pigment diffusing in the agar; reverse black, dense, circular, with irregular, fimbriate margin. Sexual and asexual morphs formed in culture. Morphology of sexual phase similar to those on the substrates.

##### Additional material examined.

THAILAND, Krabi Province, Meuang district, on dead twigs of *Barringtonia acutangula* (Lecythidaceae), 16 December 2015, C. Phukhamsakda, Kr015, (**isotype in HKAS**, under the code of HKAS 99512).

##### Distribution.

Krabi Province, Thailand

##### Notes.

**Figure 3.**
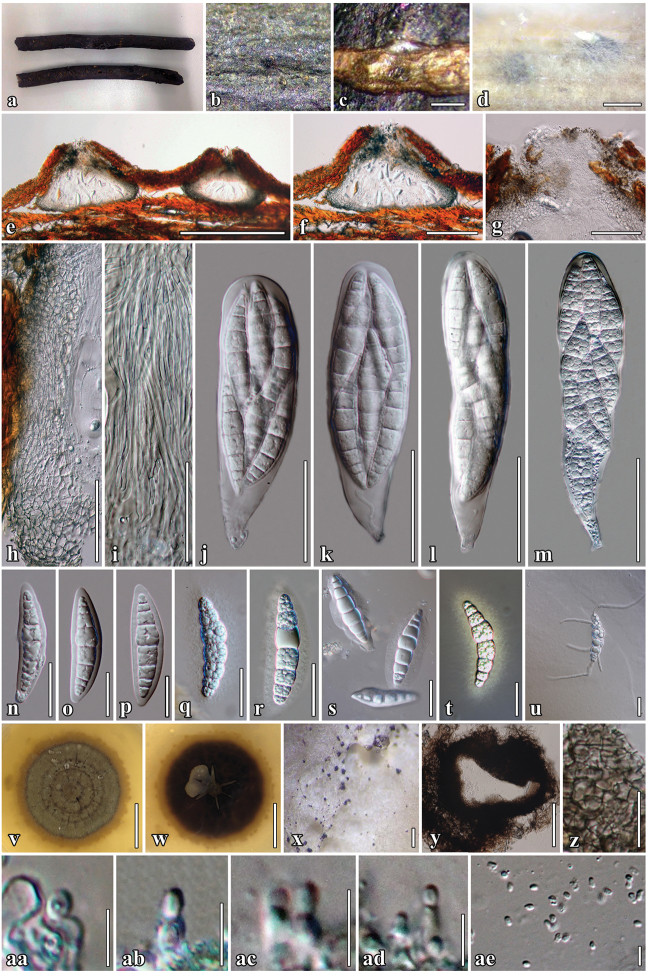
***Neoaquastroma krabiense*** (MFLU 17-0003, holotype) **a** *Barringtonia acutangula* (L.) Gaertn specimens **b** Appearance of ascomata on host surface **c** Close up of ascomata **d** Ascomata forming on rice straw on WA after 8 weeks **e, f** Section of ascoma **g** Ostiolar canal **h** Section of partial peridium layer **i** Hyaline pseudoparaphyses **j–m** Asci **n–s** Hyaline ascospores with visible mucilaginous sheath **q** Ascospores stained in Indian ink to show sheath **u** Germinated ascospore **v, w** Culture characteristics on MEA **x, y** Conidiomata forming in culture after 8 weeks **z** Conidiomatal wall **aa–ad** Conidiogenous cells and developing conidia **ae** Conidia **n–p** Ascospores in 5% of KOH reagent **m, r** Asci and ascospore in culture (on rice straw). Scale bars: 500 µm (**c–e**); 200 µm (**f, x**); 50 µm (**g–m, y**), 20 µm (**n–u, z**); 5 µm (**aa-af**); 20 mm (**v–w**).

*Neoaquastroma krabiense* was collected in the southern part of Thailand on dead twigs of *Barringtonia acutangula*. It is placed in *Neoaquastroma* based on its morphology of both sexual and asexual morph and close phylogenetic affinity to other species of *Neoaquastroma*. *Neoaquastroma krabiense* is distinct in that it has a flattened ascomata base and larger and more slender asci and ascospores than *N. guttulatum* and *N. bauhiniae*. The species formed an asexual morph in culture (Fig. 3, m) as pycnidial conidiomata with hyaline conidia (Fig. 3, x-ae).

## Discussion

In the present study, we introduce two new species of *Neoaquastroma*, as *N. bauhiniae* and *N. krabiense*. The descriptions were made from fungi isolated from dicotyledonous plants in Thailand. The new species are introduced based on multi-locus phylogeny coupled with morphology that support their placement within Parabambusicolaceae.

Parabambusicolaceae is typified with *Parabambusicola* Tanaka & K. Hiray. The type of the genus was described originally as *Massarina bambusina* Teng (Teng, 1936) from bamboo. The family is characterised by ascomata surrounded by stromatic tissues and multiseptate, clavate to fusiform and hyaline ascospores (Tanaka and Harada 2003, Tanaka et al. 2015). The asexual morph in Parabambusicolaceae can be coelomycetous or hyphomycetous. Sporodochia or pycnidia with hyaline conidia are formed in *Parabambusicola* and *Neoaquastroma* (Tanaka et al. 2015, this study), while hyphomyceteous structures are known from *Pseudomonodictys* and *Monodictys* spp. (Ariyawansa et al. 2015, Tanaka et al. 2015).

*Neoaquastroma* was introduced as a distinct genus in Parabambusicolaceae, with *N. guttulatum* as the type species (Wanasinghe et al. 2017). The genus resembles *Parabambusicola* and *Multiseptospora*, but form distinct lineages in phylogenetic studies (Liu et al. 2015, Tanaka et al. 2015, Wanasinghe et al. 2017). *Parabambusicola* and *Neoaquastroma* are similar in their morphology. The differentiation between *Multiseptospora*, *Neoaquastroma* and *Parabambusicola* is predominantly based on the morphology of ascospores, particularly with the size and number of septa.

In the phylogenetic analyses of Wanasinghe et al. (2017), Parabambusicolaceae clustered into three clades, where *Neoaquastroma guttulatum* (MFLUCC 14-0917) clustered with *Aquastroma magniostiolata* (KT 2485), *Multilocularia bambusae* (MFLUCC 11-0180), *Monodictys* sp. (JO 10, KH 331) and *Pseudomonodictys tectonae* (MFLUCC 12-0552) with high statistical support. In this study, *Neoaquastroma* forms a separate clade, sister to *Multiseptospora* and *Parabambusicola*. This is probably due to limited taxon sampling.

## Supplementary Material

XML Treatment for Neoaquastroma bauhiniae

XML Treatment for Neoaquastroma krabiense
